# Crop rotation increases root biomass and promotes the correlation of soil dissolved carbon with the microbial community in the rhizosphere

**DOI:** 10.3389/fbioe.2022.1081647

**Published:** 2022-12-06

**Authors:** Shuaimin Chen, Fanyun Yao, Guohua Mi, Lichun Wang, Haiyan Wu, Yongjun Wang

**Affiliations:** ^1^ Institute of Agricultural Resource and Environment, Jilin Academy of Agricultural Sciences, Changchun, China; ^2^ Key Laboratory of Plant–Soil Interactions, Ministry of Education, College of Resources and Environmental Sciences, China Agricultural University, Beijing, China

**Keywords:** conservation agriculture, straw mulching, maize-peanut rotation, labile carbon fractions, root biomass

## Abstract

As essential approaches for conservation agricultural practices, straw residue retention and crop rotation have been widely used in the Mollisols of Northeast China. Soil organic carbon, root development and microbial community are important indicators representing soil, crop and microbiota, respectively, and these factors work together to influence soil fertility and crop productivity. Studying their changes and interactions under different conservation practices is crucial to provide a theoretical basis for developing rational agricultural practices. The experiment in this study was conducted using the conventional practice (continuous maize without straw retention, C) and three conservation practices, namely, continuous maize with straw mulching (CS), maize–peanut rotation (R), and maize–peanut rotation with straw mulching (RS). Straw mulching (CS) significantly increased soil total organic carbon (TOC), active organic carbon (AOC), and microbial biomass carbon (MBC), but did not promote maize yield. Maize–peanut rotation (R and RS) significantly increased dissolved organic carbon (DOC) in the rhizosphere by promoting root growth, and maize yield (increased by 10.2%). For the microbial community structure, PERMANOVA and PCoA indicated that the bacterial community differed significantly between rhizosphere soil and bulk soil, but the fungal community shifted more under different agricultural practices. The correlation analysis indicated that the rotation system promoted the association between the soil DOC and the microbial community (especially the bacterial community), and straw mulching enhanced the connection between the soil TOC and the fungal community. Some plant growth–promoting rhizobacteria (including *Bacillus*, *Streptomyces*, *Rhizobium*, and *Pseudomonas*) were enriched in the rhizosphere soil and were increased in the rotation system (R and RS), which might be due to an increase in the soil rhizosphere DOC level. These beneficial microbes had significantly negative correlations with several fungal groups (such as *Mycosphaerella*, *Penicillium*, *Paraphoma* and *Torula*) that were classified as plant pathotrophs by FUNGuild. These results indicated that ensuring plant root development and improving root–bacteria interactions are of great importance to guarantee crop yield when implementing conservation tillage practices.

## Introduction

Food security, given the rapid increase in the human population and the degradation of cultivated land resources, is a global concern. High–intensity conventional tillage, fertilisation and pesticides substantially promote crop production. However, these practices have also introduced a number of negative effects, including soil biodiversity reduction, soil quality degradation, and soil erosion magnification, further decreasing soil fertility (Okada et al., 2014; Sliva et al., 2014; Tsiafouli et al., 2015; Bender et al., 2016). These changes have severely restricted agricultural sustainability. Northeast China is one of the major crop production regions in the country, and during the past few decades, its production of maize (*Zea mays* L.) contributed to nearly 1/3 of China’s total forage and food supply (Zhao et al., 2015). However, long–term soil overuse has caused a series of issues, such as soil erosion, soil structure deterioration, and a decline in soil fertility (Yu et al., 2006; Liu et al., 2009).

Conservation agriculture constitutes basic principles, including reduction in tillage, retention of adequate crop residue levels on the soil surface, and the use of crop rotation (Govaerts et al., 2009). Some agricultural practices based on these principles have been implemented in Northeast China. Considering the reuse of maize straw, straw mulching with reduced tillage is widely used. Northeast China is also a major production area for legumes such as peanuts (*Arachis hypogaea*) and soybeans (*Glycine max*). Due to market demands and policy adjustments in China, peanuts offer a better economic benefit than soybeans, and are more favoured by local farmers. Maize–peanut rotation is gradually being applied in Northeast China. However, not all conservation agricultural practices are adopted in all fields, and it is necessary to study their potentials and limits on sustainable agriculture (Pittelkow et al., 2015). The soil, crop roots and microorganisms are fundamental factors impacting agricultural productivity because of their complex interactions in the rhizospheric zone. Studying the individual changes and mutual correlations of these factors could strengthen the understanding of the effects of different agricultural practices on soil fertility and crop productivity.

Soil organic carbon (SOC) is considered an important component of soil fertility (Lal, 2004; Lal, 2006) because it is closely associated with a wide array of physical, chemical and biological characteristics and plays a key role in determining and maintaining soil physicochemical conditions and functions (Dexter et al., 2008). Furthermore, labile SOC fractions, which are variable proportions of SOC with turnover times of a few days to months, are highly sensitive indicators of changes in soil fertility and quality (Xu et al., 2011; Benbi et al., 2015; Muñoz–Romero et al., 2017). They are derived from root exudates, plant residues, and microorganisms and their metabolites, and include potassium permanganate oxidisable organic C (KMnO_4_–C, AOC), dissolved organic C (DOC), and microbial biomass C (MBC). Labile SOC fractions have therefore been extensively used to assess soil fertility under different agricultural practices. Many studies have discussed the effect of tillage, rotation, and residue management on soil carbon stocks (West and Post., 2002; Govaerts et al., 2009; Palm et al., 2014). For example, straw retention with no–tillage has been reported to be one of most effective agricultural practices for soil carbon sequestration (Shen et al., 2021). Crop rotation can promote carbon sequestration by generating a legacy effect of the temporal diversity in plants (Zhao et al., 2020). Additionally, labile SOC fractions are generally increased with residue retention (Palm et al., 2014; Bu et al., 2020). However, effects of conservation agricultural practices on crop productivity are difficult to identify clear results in different regions (Liu et al., 2019; Zhao et al., 2020).

High agricultural productivity depends not only on high soil fertility but also on optimum root growth (Hammel, 1994; Ren et al., 2018). This is because crop roots are vital organs for absorbing water and nutrients, and their growth and development directly impact aboveground growth and yield (Garnett et al., 2009; Lynch, 2013; Takatoshi and Anne, 2016). Crop roots are most sensitive to changes in soil physical and chemical properties, such as soil bulk density, penetration resistance, water and nutrient conditions (Hamblin, 1986; Dexter, 1988). The effects of agricultural practices on soil conditions and root growth have been widely studied (Dam et al., 2005; He et al., 2007; Ren et al., 2018), and it has been indicated that suitable agricultural practices can reduce soil penetration resistance to increase root length density and dry matter, and improve the soil movement of air and water to enhance root activity. Thus, the effects of agricultural practices on soil conditions can be reflected on crop roots.

Soil microorganisms are essential to sustain soil fertility because of their irreplaceable roles in biogeochemical cycles and crop nutrient uptake. It is generally considered that conservation agricultural practices promote higher microbial diversity and biomass because of less soil disturbance and more carbon/nutrient input (Wang et al., 2017; Wang et al., 2020), but these effects remain controversial in various soil conditions. Undoubtedly, soil microbial communities and functions are strongly influenced by agricultural practices due to changes in the quantity and quality of crop residues, and changes in the physical and chemical soil conditions (Sun et al., 2018; Cong et al., 2020). These changes would also alter the community assembly and ecosystem function of rhizosphere microbiota. The rhizosphere is the zone surrounding plant roots where complex interactions take place between roots and microorganisms (Philippot et al., 2013). The rhizospheric microbiota utilises a broad variety of chemical compounds (rhizodeposits) released by plants, and its diversity and activity are also regulated by rhizodeposits (e.g., root exudates, border cells, mucilage). The microbiota can be beneficial or harmful to the host plant, and a shift in this balance might substantially affect crop productivity (Mendes et al., 2013). Most soil–borne pathogens are saprophytic and need to reach sufficient numbers on their host before they infect host tissues. Rhizodepositions can absorb beneficial rhizobacteria, such as plant growth–promoting rhizobacteria (PGPR) and biocontrol microorganisms, to resist pathogen infection and enhance environmental adaptability (Dutta and Podile, 2010; Hu et al., 2018). Therefore, understanding how rhizospheric microbial communities respond to agricultural practices is of great agronomic interest.

Soil organic carbon, root development and the microbial community are important indicators representing soil, crop and microbiota, respectively. Studying their changes and interactions under different conservation practices is crucial to elucidate the key factors limiting crop productivity and to provide a theoretical basis for developing rational agricultural practices. In this study, our objectives were to address the following questions: 1) Does increasing the SOC content certainly promote the crop yield? 2) What are the key factors impacting the crop productivity in different conservation agricultural practices?

## Materials and methods

### Field experiment

The maize–peanut rotation field experiment started in 2015 at the Halahai Agroecosystem Experimental Station in Nong’an County, Jilin Province, China (44°05′N, 124°51′E). There were four agricultural practice treatments: 1) conventional practice (C), where maize was continuously planted without residue retention; 2) continuous maize cropping with straw mulching (CS); 3) maize–peanut rotation without straw retention (R); and 4) maize–peanut rotation with straw mulching (RS). Considering the operability of agricultural management, the experiments were performed using a split–plot design. Continuous and rotational cropping were applied to the main plots, rotation was adopted for 1–year maize and 1–year peanut, and all peanut straw (approximately 2,000 kg/ha) was returned to the field. Maize straw retention (0%, 30%, 60%, and 100%) was employed for the subplots through a random arrangement with triplicate plots (120 m^2^ per plot). Specifically, this study selected 0% and 60% (approximately 6,000 kg/ha) retention amounts. After harvesting the maize in October, the straw was mechanically crushed and mulched on the soil surface. A strip–till machine was used to prepare a straw–free seeding belt for sowing in early May of the following year. The planting density of maize was 64,000 plants/ha. The application rate of the base fertiliser was 145 N kg/ha, 145 P_2_O_5_ kg/ha, and 145 K_2_O kg/ha, and an additional 75 N kg/ha was applied at the 11th leaf stage of maize. The planting density of peanut was 120,000 plants/ha. The base fertiliser application rate was 86 N kg/ha, 122 P_2_O_5_ kg/ha, and 115 K_2_O kg/ha when planting the peanut in R and RS.

### Maize biomass and grain yield

Plants were collected at the tassel stage (VT) for each treatment on 25 July 2020. Three maize plants were randomly selected from each plot (three plots in each treatment). The aboveground parts were cut into small pieces and dried at 80°C to constant weight. To obtain the root samples, a soil column with a diameter of 30 cm and a depth of 20 cm was excavated with the base of the maize stalk acting as the centre. After excavation, the roots were washed in water to remove the soil. The root samples were then oven–dried at 80°C to constant weight. At the physiological maturity stage (5 October), the maize grain yield (14% grain moisture content) was measured within an area of two central rows (65 cm wide × 20 m long) in each plot.

### Soil sample collection

Bulk and rhizosphere soil samples for maize were collected at the 10th leaf (V10), tassel (VT), and dough stage (R4) for each treatment on 3 July, 25 July, and 20 August in 2020, respectively. Bulk soil samples were randomly collected from three cores (4.3 cm in diameter) in each plot at 0–20 cm and homogenised into one replicate. Rhizosphere soil samples were defined as the soil attached tightly to the root surface (within 2 mm). Three plants were randomly collected from each plot. After shaking the roots to remove loosely attached soil, the rhizosphere soil samples were brushed and homogenised into one replicate. Soil samples were collected in triplicate for each treatment. Soil samples were sieved using a 2-mm filter to eliminate plant residues, stones, and other impurities, separated into two parts and stored at 4°C and −80°C for chemical analysis and DNA extraction, respectively.

### Determination of total organic carbon, active organic carbon, dissolved organic carbon, and microbial biomass carbon

Soil total organic carbon (TOC), active organic carbon (AOC), dissolved organic carbon (DOC), and microbial biomass carbon (MBC) in both rhizosphere and bulk soils were measured to assess the effects of agricultural practices and plant growth stages on soil carbon. Soil TOC was measured using the K_2_Cr_2_O_7_–H_2_SO_4_ oxidation method (Nelson and Sommers, 1982). Soil AOC was measured using the potassium permanganate (KMnO_4_) oxidation method. Exactly 1.00 g of air–dried soil was mixed with 20 ml of 0.02 M KMnO_4_ and shaken at 200 rpm at 25°C for 2 min. Subsequently, the samples were centrifuged at 950 *g* for 5 min, and 1 ml of the supernatant was pipetted and diluted with deionized water to 50 ml. An ultraviolet spectrophotometer (UV-2450, Shimadzu) was used to measure the absorbance of the diluted samples at 550 nm. The amount of MnO_4_^−^ consumed was calculated from the difference with the blank group (no soil). The reduction of 1 mM MnO_4_^−^ is equivalent to the oxidation of 0.75 mM or 9 mg of carbon (Blair et al., 1995). Soil DOC was extracted by agitating 10 g of soil with 20 ml deionized water at 25°C for 1 h. The extraction was centrifuged at 10,000 *g* for 5 min, and the supernatant was filtered through a 0.22-μm syringe filter. For DOC determination, the filtered supernatant was measured using a TOC analyser. Soil MBC was measured using the chloroform (CHCl_3_) fumigation–extraction method (Vance et al., 1987). The soil (20.00 g) was fumigated using purified liquid chloroform for 24 h. The soil was then extracted with 0.5 mol/L K_2_SO_4_ (1:4 soil:extractant) for 30 min. The unfumigated soil samples were also extracted. After filtration, the filtrate was used to establish the concentration of soluble organic carbon in the soil using a Liqui TOCII analyser (Elementar, Germany). The mass fraction of soil MBC was calculated using the formula MBC = *E*_*C*_/*K*_*EC*_, where E_C_ is the difference between the fumigated and unfumigated soil samples (K_EC_ = 0.45).

### Soil DNA extraction, amplicon sequencing, and bioinformatics analysis

Total soil DNA was extracted using a Fast^®^ DNA SPIN Kit (MP Biomedicals, Santa Ana, CA, United States) following the manufacturer’s protocol. The primer pairs 338F/806R (5′–ACTCCTACGGGAGGCAGCA–3′; 5′–GGACTACHVGGGTWTCTA AT–3′) (Mori et al., 2014) and ITS5F/ITS1R (5′–GGA AGT​AAA​AGT​CGT​AAC​AAG​G–3′; 5′–GCT​GCG​TTC​TTC​ATC​GAT​GC–3′) (White et al., 1990) were used to amplify the V3–V4 region of the bacterial 16S rRNA gene and region 1 of the fungal ITS gene. Sequencing was performed using the Illumina NovaSeq-PE250 platform (Illumina, San Diego, CA, United States) at Shanghai Personal Biotechnology Co., Ltd., and the sequencing data were analysed using QIIME2-2019.4 (Bolyen et al., 2019). Raw sequence data were demultiplexed *via* q2-demux. The sequences were then quality–filtered, deionised, and merged, and the chimaeras were removed using DADA2 (*via* q2-dada2) (Callahan et al., 2016). Non-singleton amplicon sequence variants (ASVs) were aligned with MAFFT (*via* q2-alignment) (Katoh et al., 2002). Overall, 45,601–113,681 clean 16S rRNA gene sequences per sample and 65,619–146,894 clean ITS sequences per sample were obtained. For downstream analysis, all samples were rarefied to 43,320 16S rRNA gene sequences and 54,546 ITS gene sequences. The sequencing depth was adequate because the good coverage sequences in all samples were above 96%. Taxonomy was assigned to ASVs using the classify–sklearn naive Bayes taxonomy classifier (*via* q2-feature–classifier) (Bokulich et al., 2018). After rarefying the samples, the alpha diversity metric (observed species) and beta diversity metric (Bray‒Curtis distance) were estimated using the q2-diversity plugin. SILVA (release 132) and UNITE (release 8.0) were used as reference databases for 16S rRNA and ITS genes, respectively. Microbial community compositions were illustrated at the family and genus levels. The ecological guilds of the fungal operational taxonomic units (OTUs) were predicted using FUNGuild (Nguyen et al., 2016). Sequencing data were deposited in the NCBI under the accession number PRJNA776676.

### Statistical analysis

Statistical analyses were performed in IBM SPSS Statistics (Windows 24.0). One–way ANOVA followed by Duncan’s multiple range analysis (*p* < 0.05) was performed to test the significance of the effect of agricultural practices on root weight and grain yield. Two–way ANOVA followed by Duncan’s multiple range analysis (*p* < 0.05) was performed to test the significance of the effects of agricultural practices and plant growth stages on soil organic carbon, as well as bacterial and fungal observed species. Principal coordinate analysis (PCoA) and permutational multivariate analysis of variance (PERMANOVA) for microbial community structures were performed based on the Bray‒Curtis distance (ASV level). The Mantel test was performed using the Spearman correlation method in the vegan library in R (Oksanen et al., 2013). The soil TOC, AOC, DOC and MBC were calculated based on the Euclidean distance, and microbial community structures (ASV level) were calculated based on the Bray‒Curtis distance. Spearman correlation analysis between microbial groups and organic carbon fractions was performed using the psych package in R software (Revelle, 2018). At the family level, to clearly visualise the variations in relative abundance among different treatments, the normalised relative abundance of a soil sample (NRA_i_) was calculated using the formula NRA_i_ = l g (RA_i_/RA_bulk-V10-C_), where RA_i_ is the relative abundance of a soil sample and RA_bulk-V10-C_ is the relative abundance in the C treatment at the V10 stage for bulk soil.

## Results

### Maize biomass and yield

The root biomass, shoot biomass, and root–shoot ratio at the VT stage were shown in Table 1. The root biomass in the rotation systems (23.5 g/plant and 23.2 g/plant in R and RS, respectively) was significantly greater than that in the conventional practice (C, 19.3 g/plant). The root biomass in CS was 16.5 g/plant, which was the smallest. The shoot biomass in the straw mulching treatments (182 g/plant and 178 g/plant in CS and RS, respectively) was significantly greater than that in the no–straw retention treatments (164 g/plant and 170 g/plant in C and R, respectively). The root–shoot ratio in CS was the smallest compared with that in the other treatments. For the grain yield (Table 1), compared with the conventional practice (C, 11.7 × 10^3^ kg/ha), the yield was the smallest in CS (10.9 × 10^3^ kg/ha) and was the greatest in the rotation systems (12.9 × 10^3^ kg/ha in the R and RS). The root biomass (*r* = 0.75) and root–shoot ratio (*r* = 0.75) had significantly positive correlations with maize yield.

**TABLE 1:** Maize biomass at the VT stage (the tassel stage) and grain yield under different agricultural practices.

Agricultural practices	Root biomass (g/plant)	Shoot biomass (g/plant)	Root-shoot ratio	Grain yield (×10^3^ kg/ha)
C	19.3 ± 1.4b	164 ± 5c	0.12 ± 0.02a	11.7 ± 0.5b
CS	16.5 ± 1.5c	182 ± 6a	0.07 ± 0.01b	10.9 ± 0.3c
R	23.5 ± 1.1a	170 ± 6bc	0.14 ± 0.05a	12.9 ± 0.2a
RS	23.2 ± 1.3a	178 ± 8ab	0.14 ± 0.03a	12.7 ± 0.3a

C: continuous maize cropping without straw retention; CS: continuous maize cropping with straw mulching; R: maize-peanut rotation without straw retention; RS: maize-peanut rotation with straw mulching.

Different letters indicate significant difference (*p* < 0.05) under different treatments by one-way ANOVA.

### Soil organic carbon fractions

**FIGURE 1:**
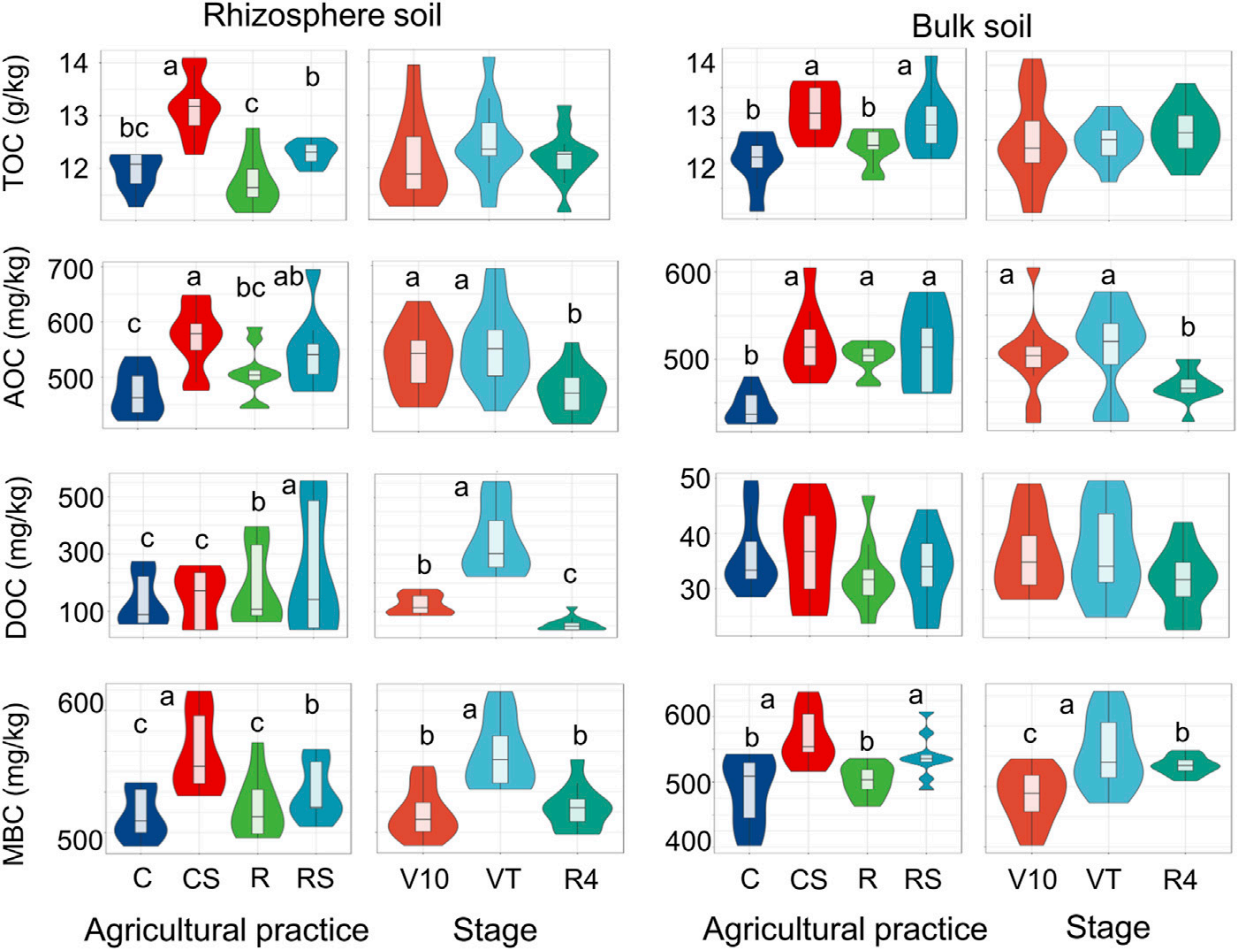
Soil organic carbon fractions under different agriculture practices and at different plant growth stages. Different letters indicate significant differences (*p* < 0.05) under different treatments by one-way ANOVA. C: continuous maize cropping without straw retention; CS: continuous maize cropping with straw mulching; R: maize-peanut rotation without straw retention; RS: maize-peanut rotation with straw mulching; V10: the 10th leaf stage; VT: tassel stage; R4: dough stage; TOC, total organic carbon; AOC, active organic carbon; DOC, dissolved organic carbon; MBC, microbial biomass carbon.

Soil organic carbon fractions were analysed according to agricultural practice and maize growth stage (Figure 1; Supplementary Table S1 in the Supplementary Material S1). Additionally, the levels of these soil carbon fractions under four agricultural practices at three growth stages are listed in Supplementary Figure S1. Straw mulching significantly increased the soil TOC. The soil TOC level was 11.8–14.1 g/kg, 11.6–13.6 g/kg, 11.1–12.7 g/kg, and 10.5–12.3 g/kg in CS, RS, R, and C, respectively. The soil AOC and MBC were significantly affected by agricultural practices and plant growth stages. They were significantly increased by straw mulching. The soil AOC at the V10 and VT stages was significantly greater than that at the R4 stage, whereas the soil MBC at the VT stage was significantly greater than that at the V10 and R4 stages. The level of DOC in the rhizosphere soil samples was significantly influenced by agricultural practices and plant growth stages. The DOC level at the VT stage (222–556 mg/kg) was significantly greater than that at the V10 and R4 stages (87–178 mg/kg and 34–116 mg/kg, respectively). At the VT stage, the mean DOC in the rotation systems (364 mg/kg and 523 mg/kg in R and RS, respectively) was significantly greater than that in the continuous systems (251 mg/kg and 248 mg/kg in C and CS, respectively) (Supplementary Figure S1C in the Supplementary Material S1).

### Soil microbial communities

**FIGURE 2:**
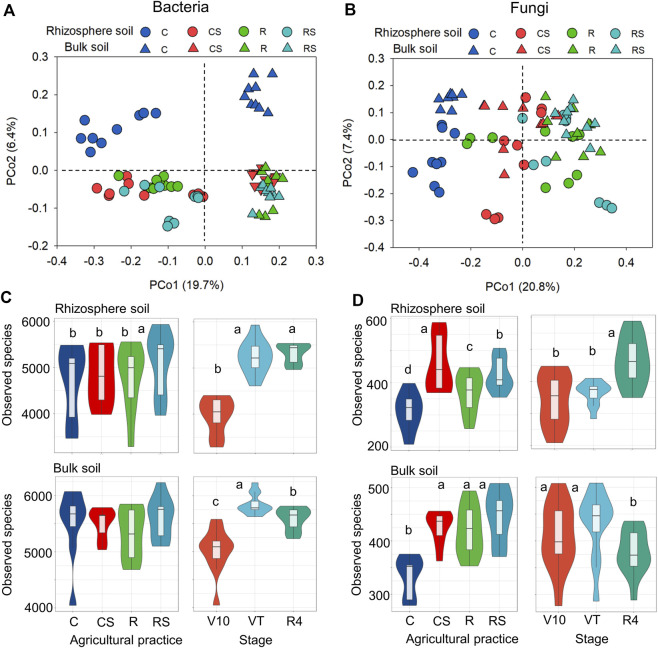
Soil microbial communities under different agricultural practices and at different plant growth stages. PCoA of the bacterial community **(A)** and fungal community **(B)**. Identified bacterial **(C)** and fungal **(D)** species. Different letters indicate significant differences (*p* < 0.05) under different treatments by one-way ANOVA for 2C and 2D. C: continuous maize cropping without straw retention; CS: continuous maize cropping with straw mulching; R: maize-peanut rotation without straw retention; RS: maize-peanut rotation with straw mulching; V10: the 10th leaf stage; VT: tassel stage; R4: dough stage.

Based on the Bray‒Curtis dissimilarity matrix, PERMANOVA (Table 2) was performed to assess the effects of agricultural practices and plant growth stages on microbial community structures. When PERMANOVA was performed using total soil samples, the bacterial community structure was significantly different between rhizosphere soil and bulk soil (17.4% explained variance), while the fungal community structure was not significantly different. Agricultural practices had greater effects on both bacterial and fungal community structures (10.7% and 26.9%, respectively) than plant growth stages (7.1% and 8.1%, respectively). When PERMANOVA was executed using rhizosphere or bulk soil samples, only the plant growth stage had no significant effect on the fungal community in bulk soil. The PCoA plots also showed the variations in microbial communities in the different treatments (Figures 2A,B). For bacteria, the plots were separated by rhizosphere and bulk soil along axis 1 (19.7%) and agricultural practice C was separated from the other agricultural practices (CS, R, and RS) along axis 2 (6.4%). For fungi, the plots were separated by different agricultural practices along axis 1 (20.8%) for fungi. In addition, the PCoA plots representing different treatments (agricultural practices and maize growth stages) in rhizosphere soil and bulk soil are shown in Supplementary Figure S2 (Supplementary Material S1). The observed species in the samples were calculated to evaluate the effects of agricultural practices and plant growth stages on microbial diversity (Figures 2C,D). The results showed that agricultural practices had a greater effect on fungal diversity, while plant growth stages had a greater effect on bacterial diversity (Supplementary Table S1 in the Supplementary Material S1).

**TABLE 2:** PERMANOVA of microbial community structures receiving different treatments.

Factors	Explained variance			
		Total (*n* = 72)	Rhizosphere (*n* = 36)	Bulk (*n* = 36)
Bacteria	AP	10.7%**	18.3%**	18.1%**
	S	7.1%*	18.9%**	10.5%**
	L	17.4%**		
Fungi	AP	26.9%**	33.2%**	32.05%**
	S	8.1%*	21.0%**	ns
	L	ns		

** PERMANOVAs are based on the Bray-Curtis dissimilarity matrix. The significance level, ns, not significant; *p < 0.05; *p* < 0.01.

AP, agriculture practices (C, CS, R and RS); S, plant growth stages (V10, VT, and R4); L, locations (rhizosphere and bulk). C: continuous maize cropping without straw retention; CS: continuous maize cropping with straw mulching; R: maize-peanut rotation without straw retention; RS: maize-peanut rotation with straw mulching; V10, the 10th leaf stage; VT: tassel stage; R4: dough stage.

### Soil microbial taxa

**FIGURE 3:**
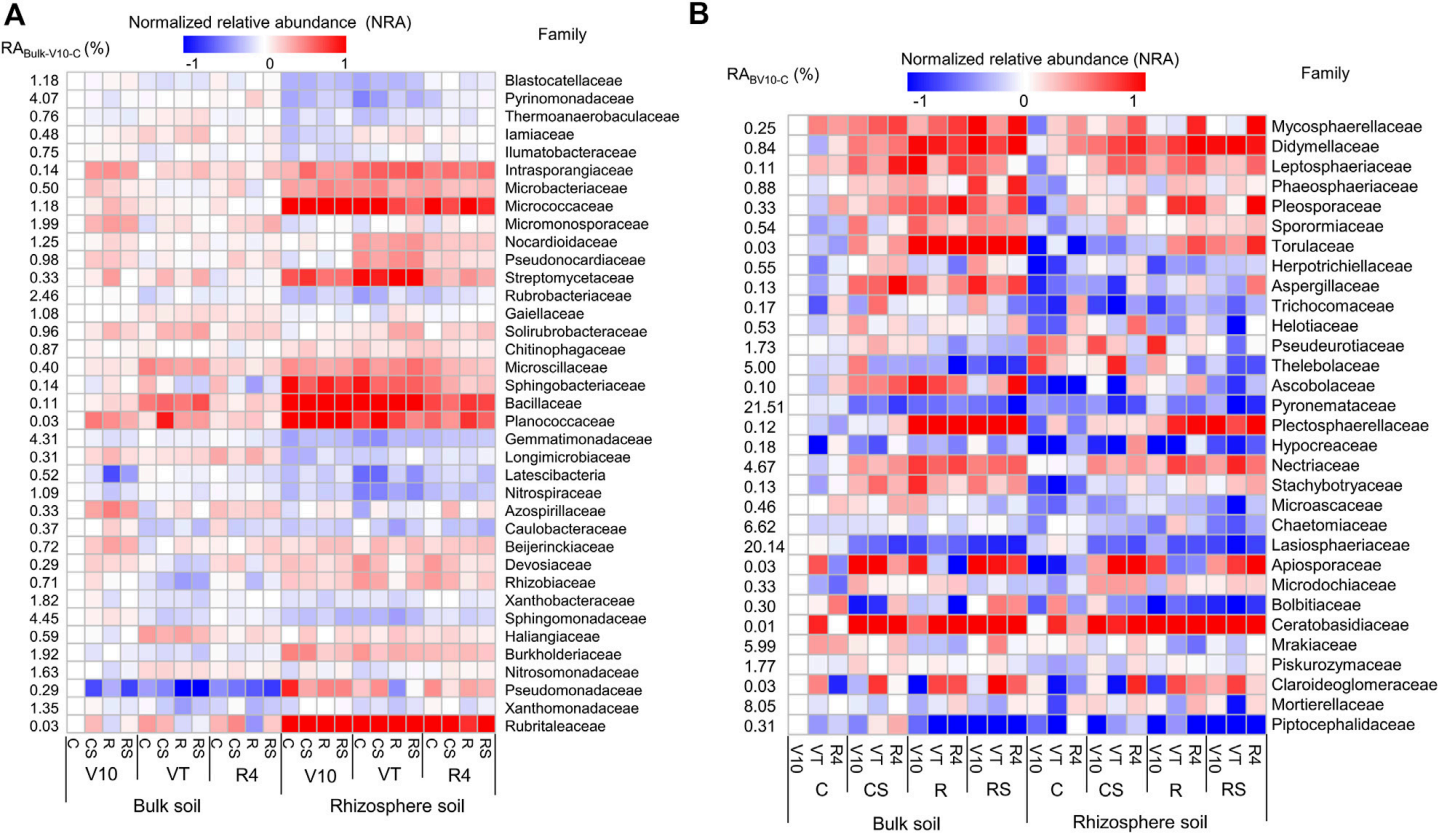
Normalised relative abundance of bacterial **(A)** and fungal **(B)** families in different treatments. The normalised relative abundance of a soil sample (NRA_i_) was calculated using the formula NRA_i_ = lg (RA_i_/RA_bulk-V10-C_), where RA_i_ is the relative abundance of a soil sample, and RA_bulk-V10-C_ is the relative abundance of the C treatment at the V10 stage for the bulk soil. C: continuous maize cropping without straw retention; CS: continuous maize cropping with straw mulching; R: maize-peanut rotation without straw retention; RS: maize-peanut rotation with straw mulching; V10: the 10th leaf stage; VT: tassel stage; R4: dough stage.

At the family taxonomic level, families with the relative abundance greater than 0.5% were selected (37 bacterial and 31 fungal families, Supplementary Materials S2, S3). The normalised relative abundance [NRA_i_ = lg (RA_i_/RA_bulk-V10-C_)] was calculated to visualise the variations in different samples (Figure 3). The bacterial community composition showed remarkable differences between the rhizosphere and bulk soils. The relative abundances of *Intrasporangiaceae*, *Micrococcaceae*, *Nocardioidaceae*, *Streptomycetaceae*, *Sphingobacteriaceae*, *Bacillaceae*, *Planococcaceae*, *Devosiaceae*, *Rhizobiaceae*, *Burkholderiaceae*, *Pseudomonadaceae*, and *Rubritaleaceae* were significantly higher in rhizosphere soil than in bulk soil. In the rhizosphere soil, the relative abundances of *Sphingobacteriaceae*, *Bacillaceae,* and *Planococcaceae* were significantly higher at the maize V10 and VT stages than at the R4 stage, whereas the relative abundances of *Intrasporangiaceae*, *Microbacteriaceae*, *Micrococcaceae*, *Nocardioidaceae*, *Pseudonocardiaceae*, and *Streptomycetaceae* were significantly greater at the VT stage. The fungal community composition showed apparent differences among the four agricultural practices. The relative abundances of *Didymellaceae*, *Sporormiaceae*, and *Apiosporaceae* were significantly greater in agricultural practices with straw mulching (CS and RS) than in those without straw retention (C and R). The relative abundances of *Didymellaceae*, *Sporormiaceae*, *Torulaceae*, *Plectosphaerellaceae*, and *Nectriaceae* were significantly higher in the rotation systems (R and RS) than in the continuous systems (C and CS).

**FIGURE 4:**
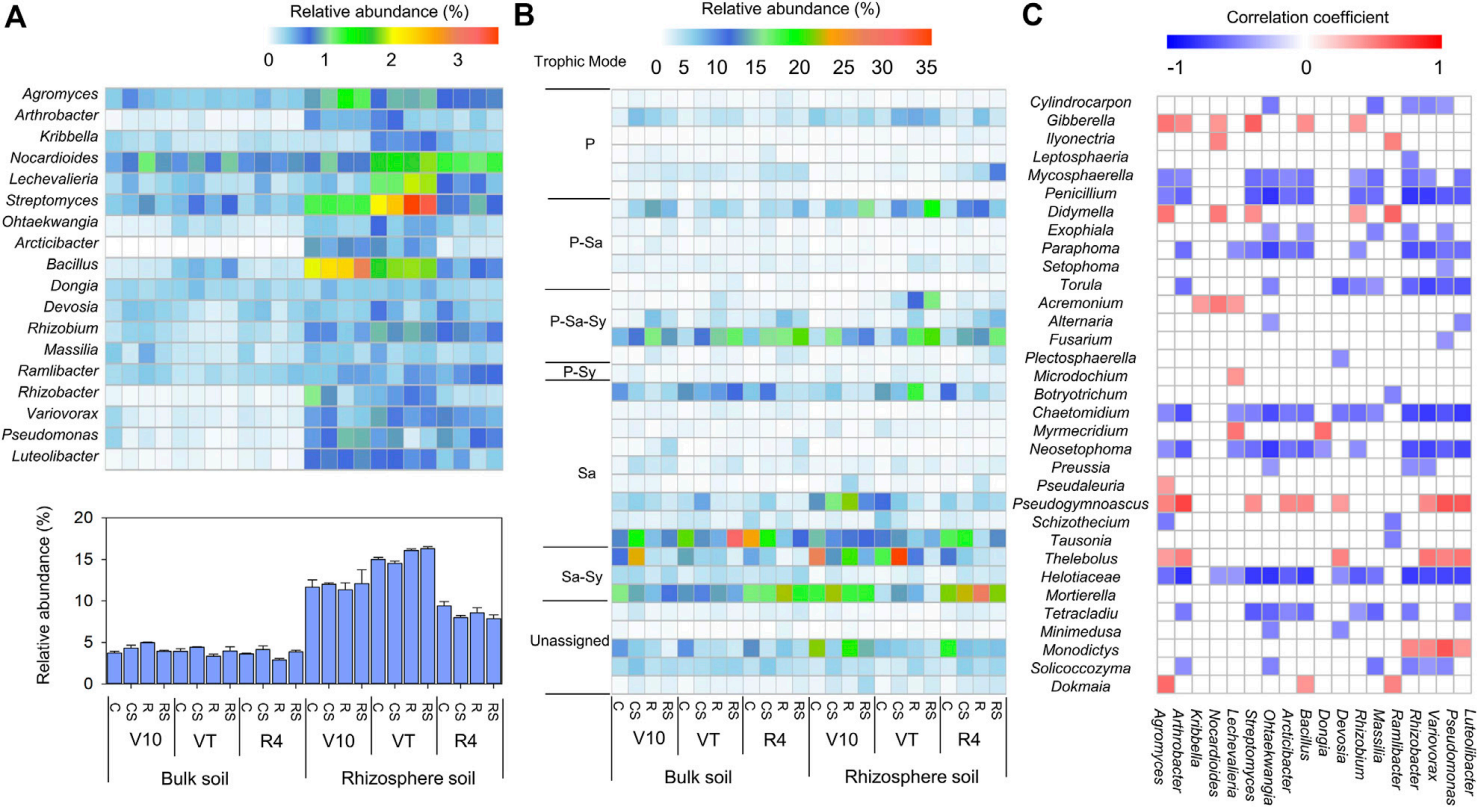
Composition of the bacterial community at the genus taxonomic level **(A)**: Heatmap showing the relative abundance of classified bacterial genera that were greater in the rhizosphere soil than in the bulk soil. In addition, the histogram shows the sum of the relative abundances of these genera in a sample. Classified fungal genera with relative abundance >0.5% **(B)** and trophic mode predicted using FUNGuild; P, pathotroph; Sa, saprotroph; Sy, symbiotroph. Correlation pattern of the bacterial genera and fungal genera **(C)**. Positive correlations (red squares) and negative correlations (blue squares) are displayed (*p* < 0.05). C: continuous maize cropping without straw retention; CS: continuous maize cropping with straw mulching; R: maize-peanut rotation without straw retention; RS: maize-peanut rotation with straw mulching; V10: the 10th leaf stage; VT: tassel stage; R4: dough stage.

At the genus level, the classified bacterial genera whose relative abundances (>0.5%) were higher in rhizosphere soil than in bulk soil were shown in Figure 4A. In the rhizosphere soil, the relative abundances of *Arcticibacter* and *Bacillus* were significantly greater at the V10 stage, while the relative abundances of *Kribbella*, *Nocardioides*, *Lechevalieria*, and *Streptomyces* were significantly greater at the VT stage. The sum of these classified genera was significantly higher at the VT stage than at the other stages. The classified fungal genera (relative abundance >0.5%) are shown in Figure 4B, and their trophic modes (pathotroph, saprotroph, and symbiotroph) were predicted by FUNGuild (Supplementary Table S2 in Supplementary Material S1). Correlations between bacterial genera and fungal genera were assessed using the total samples (Figure 4C). *Cylindrocarpon*, *Mycosphaerella*, *Penicillium*, *Paraphoma*, *Torula*, *Chaetomidium*, *Neosetophoma*, *Helotiaceae*, *and Tetracladium* were negatively correlated with many bacterial genera. Most of these fungal genera were predicted to be plant pathogens.

### Correlations between soil organic carbon fractions and microbial communities

The Mantel test was used to analyse the correlations between soil carbon fractions and microbial communities (at the ASV level) in different agricultural practices (Table 3). In the C treatment, all carbon fractions had significant correlations with the bacterial community. The soil DOC also had a significant correlation with the fungal community. Straw mulching (CS) increased the correlation between the soil TOC and the microbial community (*r* = 0.29 and *r* = 0.19, respectively, in the bacterial and fungal communities, *p* < 0.05). In the R treatment, the soil DOC and MBC were significantly correlated with the bacterial community. In the RS treatment, the soil TOC and DOC were significantly correlated with the bacterial and fungal communities.

**TABLE 3:** Correlation between soil organic carbon and microbial community structures under different agriculture practices, as determined by the Mantel test (*r* value).

Soil carbons	Bacterial community				Fungal community			
	C	CS	R	RS	C	CS	R	RS
TOC	0.26**	0.29**	ns	0.26**	ns	0.19*	ns	0.32**
AOC	0.22*	0.19*	ns	ns	ns	ns	ns	ns
DOC	0.38**	0.31**	0.22*	0.37**	0.23*	ns	ns	0.40**
MBC	0.31**	ns	0.20*	ns	ns	ns	ns	ns

The significance level, ns, not significant; *p < 0.05; **p < 0.01.

C: continuous maize cropping without straw retention; CS: continuous maize cropping with straw mulching; R: maize-peanut rotation without straw retention; RS: maize-peanut rotation with straw mulching. TOC, total organic carbon; AOC, active organic carbon; DOC, dissolved organic carbon; MBC, microbial biomass carbon.

**FIGURE 5:**
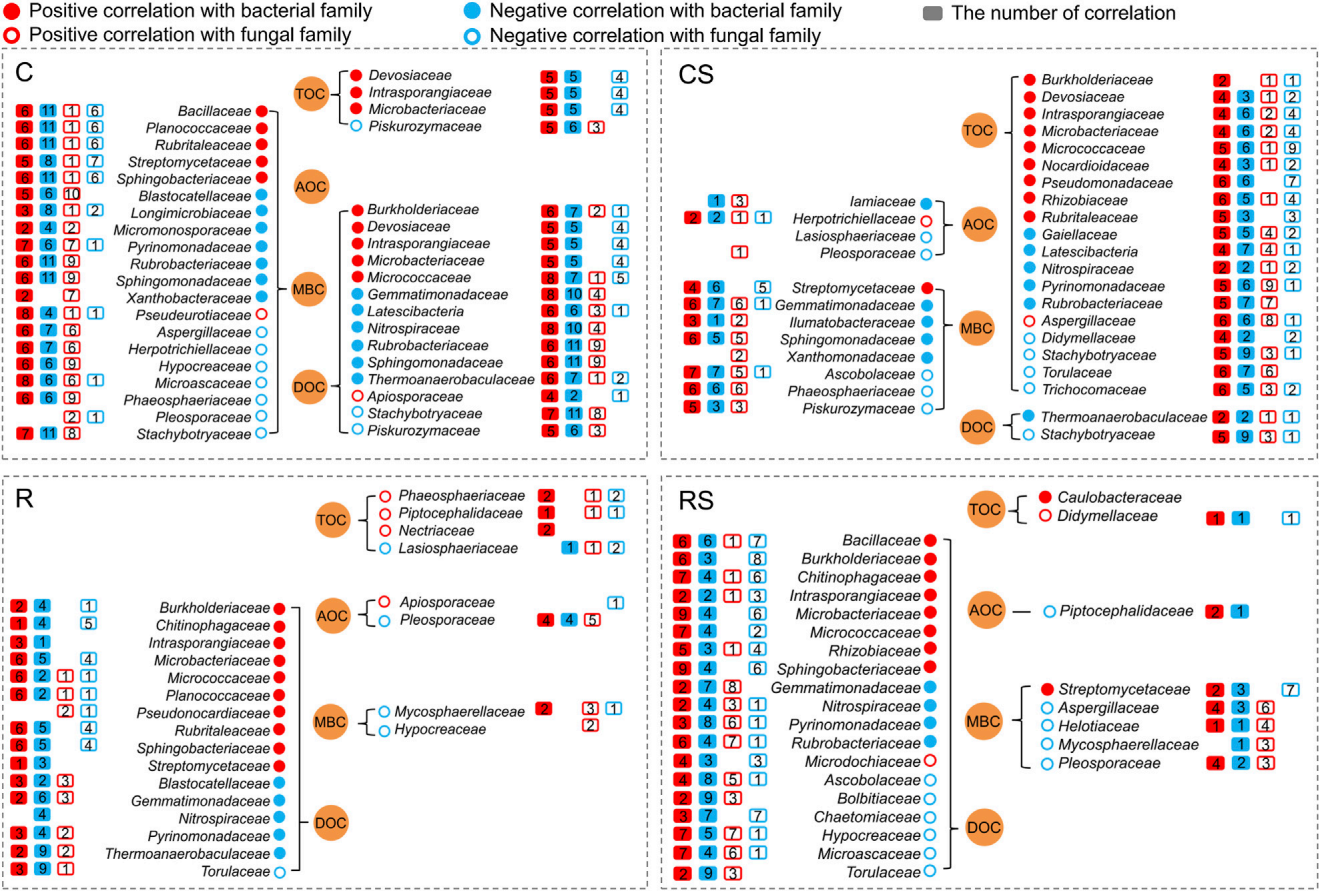
Correlations between soil carbon fractions and microbial families (*p* < 0.05). Red plots indicate positive correlation; blue plots indicate negative correlation; solid plots indicate correlation with bacterial family; blank plots indicate correlation with fungal family. The number in the box plot indicates the number of correlations between the known family and other bacterial or fungal families. For example, *Bacillaceae* was positively correlated with six bacterial families, negatively correlated with 11 bacterial families, positively correlated with one fungal family, and negatively correlated with six fungal families under the C treatment. C: continuous maize cropping without straw retention; CS: continuous maize cropping with straw mulching; R: maize-peanut rotation without straw retention; RS: maize-peanut rotation with straw mulching.

Spearman correlation analysis identified the number of correlations (*p* < 0.05) between soil carbon fractions and specific microbial groups at the family level (Figure 5). In the C treatment, soil MBC and DOC were correlated (including positively and negatively) with 20 families and 14 families, respectively. In the CS treatment, the soil TOC was correlated (including positively and negatively) with 19 families. In the R and RS treatments, the soil DOC was correlated (including positively and negatively) with 16 families and 19 families, respectively. *Bacillaceae*, *Burkholderiaceae*, *Chitinophagaceae*, *Devosiaceae*, *Intrasporangiaceae*, *Microbacteriaceae*, *Micrococcaceae*, *Sphingobacteriaceae*, and *Streptomycetaceae* were a group of families with significantly positive correlations with SOC fractions, and they also had complex interactions with other microbial families.

## Discussion

Straw mulching with no–tillage or reduced tillage has been shown to be an effective means of protecting soils. Straw mulching can control soil erosion (de Freitas and Landers, 2014; Turmel et al., 2015) and promote soil aggregation (Sheehy et al., 2015; Shen et al., 2021). Straw is an exogenous organic matter that enters the soil and is transformed to SOC through a complex decomposition process. In this study, straw mulching significantly increased the levels of soil TOC and AOC. Compared with maize residues, legume crop residues (e.g. soybean and peanut) have a low C/N ratio and are easily transformed into labile fractions (Martens, 2000). During straw decomposition, a large amount of energy sources and nutrients are released and are utilised by microbes, which might have increased the level of soil MBC in CS and RS. Furthermore, straw mulching carried large numbers of microorganisms into the soil and increased the level of MBC.

Root systems are important for water and mineral nutrient absorption from the soil to plant shoots, and are very closely related to aboveground growth and yield formation (Peng et al., 2010; Yan et al., 2011). In this study, root biomass had a significantly positive correlation with maize yield. Maize-peanut rotation systems (R and RS) could improve root biomass and maize yield. Although straw mulching with no–tillage (CS) had the highest shoot biomass, it had a negative effect on root biomass and maize yield (Table 1). Generally, aboveground growth has an important contribution to grain yield, but the contribution rate is influenced by climate, cultivar, soil fertility, and so on (Liu et al., 2020). Straw mulching would also change the morphological and physiological characteristics of roots, as well as the water condition and nutrient content in the surface soil. These changes might influence the assimilation and nutrient allocation between stems and ears.

Agricultural practices modify soil physical properties by changing the soil bulk density (Dam et al., 2005), pore size (Sasal et al., 2006), and aggregate distribution (Shen et al., 2021), and these changes alter the soil compaction. Root penetrability is correlated with soil compactibility and is one of the factors that determines root growth and development (Ren et al., 2018). Some studies reported that no–tillage or reduced–tillage increased soil bulk density and penetration resistance and limited root growth (Barber, 1971; Dam et al., 2005; Mosaddeghi et al., 2009), and indicated that maize roots in tillage soil were finer and longer than those in no–tillage soil. The results of this study showed that straw mulching with reduced tillage (CS) had a negative impact on root biomass (Table 1). Additionally, it was reported that straw mulching decreases soil surface temperature at the seeding stage to inhibit root growth, and non-decomposed straw obstructs root growth (Chen et al., 2011; Zhang et al., 2012). Compared with CS, rotation with peanut (R and RS) increased root biomass. Peanut has strong root systems, and it can form biopores in soil (a type of bio–tillage). Zhang and Peng (2021) suggested that biopores could serve as pathways for water and air flow with the help of root systems. Additionally, decomposed peanut straw might provide more available nitrogen to maize roots. Beginning at the VT stage, root development is very important for generating high maize grain yield (Qi et al., 2019), and root biomass peaks during the VT stage and milk–ripe stage (Ren et al., 2018; Qi et al., 2019). The soil DOC level in the rhizosphere was observed to be highest in the VT stage compared to the V10 and R4 stages in this study, and the rotation system (R and RS) had a greater soil DOC level in the rhizosphere than the continuous systems (C and CS) (Supplementary Figure S1 in the Supplementary Material S1).

Root exudates act as substrates and signalling molecules that attract microbes and shape the microbial structure of the rhizosphere (Badri and Vivanco, 2009; Pausch and Kuzyakov, 2018; Chen et al., 2019) and are an important component of soil DOC in the rhizosphere. Soil DOC was closely related to the bacterial communities (Table 3), and more bacterial families correlated with soil DOC in the R and RS treatments (Figure 5). Some bacterial groups, such as *Bacillus*, *Streptomyces*, *Rhizobium*, and *Pseudomonas*, that were attracted to the rhizosphere (Figure 4A) have been reported to be important PGPR (Pravin et al., 2016) and were negatively correlated with several pathogenic fungal groups, such as *Mycosphaerella*, *Paraphoma*, *Torula*, and *Helotiaceae* (Figure 4C; Supplementary Table S2 in the Supplementary Material S1). *Streptomyces* and *Bacillus* show high production of bioactive compounds (such as antibiotics, volatile compounds, and other metabolites), which help in their role as antipathogens (Gond et al., 2015; Olanrewaju and Babalola, 2019). Moreover, the composition and quantity of root exudates change during plant growth (Gransee and Wittenmayer, 2000) and influence the microbial community in the rhizosphere (Schlemper et al., 2017; Chen et al., 2019), which was also shown in this study.

Agricultural practices are one of the main factors impacting the microbial community, and in this study, the fungal community was more sensitive to agricultural practices than the bacterial community (Figure 2B; Table 2). Fungi have more diverse enzymatic capabilities and a higher capacity for decomposing complex organic materials and are important contributors to straw decomposition and complex organic carbon turnover. Several studies reported that the SOC content was a more important parameter in determining soil fungal diversity and composition (Liu et al., 2015; Wang et al., 2016). Compared to the conventional practice (C), more organic carbon (maize or/and peanut residues) was input to the soil in the conservation practices, which increased the fungal diversity (Figure 2D). In the straw mulching treatments (CS and RS), the soil TOC showed a significant correlation with the fungal community (Table 3). Meanwhile, when crop residues were retained to the soil, it would increase the population of pathogenic fungal groups and raise the risk of crop diseases (Cook and Haglund, 1991; Wang et al., 2020). For example, some species of *Fusarium* are important stalk and root rot pathogens of maize, and the relative abundance of *Fusarium* would be increased in the residue retention soil (Govaerts et al., 2008; Wang et al., 2020). In this study, the relative abundances of *Gibberella*, *Didymella*, and *Fusarium* were also increased in the conservation agricultural practices, while complex interactions between bacteria-fungi and fungi-fungi might inhibit the disease incidence.

## Conclusion

Straw mulching with reduced tillage significantly increased the level of soil TOC but did not improve root biomass or maize yield. The increase in soil TOC enhanced the fungal diversity and the correlation with the fungal community. Maize–peanut rotation systems were able to improve soil root biomass and soil DOC in the rhizosphere, as well as the maize yield. Increased soil DOC attracted more beneficial microbes around the roots, such as *Bacillus*, *Streptomyces*, *Rhizobium*, and *Pseudomonas*. These bacteria were negatively correlated with several pathogenic fungal groups. These results suggest that crop rotation can increase root biomass and promote the correlation of soil dissolved carbon with the microbial community in the rhizosphere, thus increasing maize yield.

## Data Availability

The datasets presented in this study can be found in online repositories. The names of the repository/repositories and accession number(s) can be found below: https://www.ncbi.nlm.nih.gov/, PRJNA776676.
