# Supplementary material for: Crop rotation increases root biomass and promotes the correlation of soil dissolved carbon with the microbial community in the rhizosphere
Source: Front Bioeng Biotechnol. 2022 Dec 6;10:1081647. doi: 10.3389/fbioe.2022.1081647 (PMC9763999; doi:10.3389/fbioe.2022.1081647)
Supplement: Supplementary file 1 [file Presentation1.pdf]

Table S1 The effect of agricultural practices (AP) and plant growth stages (S) on soil carbon and microbial diversity index

Factors		η^2					
		TOC	AOC	DOC	MBC	B_Obs	F_Obs
Rhizosphere	AP	0.71 **	0.51 **	0.85 **	0.83 **	0.41 *	0.88 **
	S	ns	0.45 **	0.98 **	0.87 **	0.91 **	0.87 **
	AP×S	ns	ns	0.93 **	ns	0.50 *	0.64 **
Bulk	AP	0.47 **	0.71 **	ns	0.72 **	ns	0.74 **
	S	ns	0.49 **	ns	0.70 **	0.73 **	0.41 *
	CP×S	ns	0.46 *	0.44 *	0.44 *	ns	0.41 *

The significance level, ns: not significant; *: $p<0.05$; **: $p<0.01$. η^2 describes the percentage of variance explained in the dependent variable by a predictor variable. AP: agricultural practices (C, CS, R and RS); S: plant growth stages (V10, VT and R4). C: continuous maize cropping without straw retention; CS: continuous maize cropping with straw mulching; R: maize-peanut rotation without straw retention; RS: maize-peanut rotation with straw mulching; V10: the tenth leaf stage; VT: tassel stage; R4: dough stage; TOC: total organic carbon; AOC: active organic carbon; DOC: dissolved organic carbon; MBC: microbial biomass carbon; B_Obs: observed bacterial species; F_Obs: observed fungal species.

Table S2 The ecological guilds of the fungal genera predicted by FUNGUild

Taxon	Trophic Mode	Guild
Cylindrocarpon	Pathotroph	Plant Pathogen
Gibberella	Pathotroph	Plant Pathogen
Ilyonectria	Pathotroph	Plant Pathogen
Leptosphaeria	Pathotroph	Plant Pathogen
Mycosphaerella	Pathotroph	Plant Pathogen
Penicillium	Pathotroph	Animal Pathogen
Didymella	Pathotroph-Saprotroph	Animal Pathogen-Plant Pathogen-Undefined Saprotroph
Exophiala	Pathotroph-Saprotroph	Animal Pathogen-Undefined Saprotroph
Paraphoma	Pathotroph-Saprotroph	Fungal Parasite-Plant Pathogen-Plant Saprotroph
Setophoma	Pathotroph-Saprotroph	Fungal Parasite-Plant Pathogen-Plant Saprotroph
Torula	Pathotroph-Saprotroph	Endophyte-Plant Pathogen-Wood Saprotroph
Acremonium	Pathotroph-Saprotroph-Symbiotroph	Animal Pathogen-Endophyte-Fungal Parasite-Plant Pathogen-Wood Saprotroph
Alternaria	Pathotroph-Saprotroph-Symbiotroph	Animal Pathogen-Endophyte-Plant Pathogen-Wood Saprotroph
Fusarium	Pathotroph-Saprotroph-	Animal Pathogen-Endophyte-

	Symbiotroph	Lichen Parasite-Plant Pathogen-Soil Saprotroph-Wood Saprotroph
Plectosphaerella	Pathotroph-Saprotroph-Symbiotroph	Plant Pathogen
Microdochium	Pathotroph-Symbiotroph	Endophyte-Plant Pathogen
Botryotrichum	Saprotroph	Undefined Saprotroph
Chaetomidium	Saprotroph	Dung Saprotroph-Undefined Saprotroph
Myrmecridium	Saprotroph	Undefined Saprotroph
Neosetophoma	Saprotroph	Undefined Saprotroph
Preussia	Saprotroph	Undefined Saprotroph
Pseudaleuria	Saprotroph	Undefined Saprotroph
Pseudogymnoascus	Saprotroph	Plant Saprotroph-Wood Saprotroph
Schizothecium	Saprotroph	Undefined Saprotroph
Tausonia	Saprotroph	Undefined Saprotroph
Thelebolus	Saprotroph	Dung Saprotroph
Helotiaceae	Saprotroph-Symbiotroph	Ectomycorrhizal-Fungal Parasite-Plant Pathogen-Wood Saprotroph
Mortierella	Saprotroph-Symbiotroph	Endophyte-Litter Saprotroph-Soil Saprotroph-Undefined Saprotroph
Monodictys	-	-
Solicoccozyma	-	-
Dokmaia	-	-

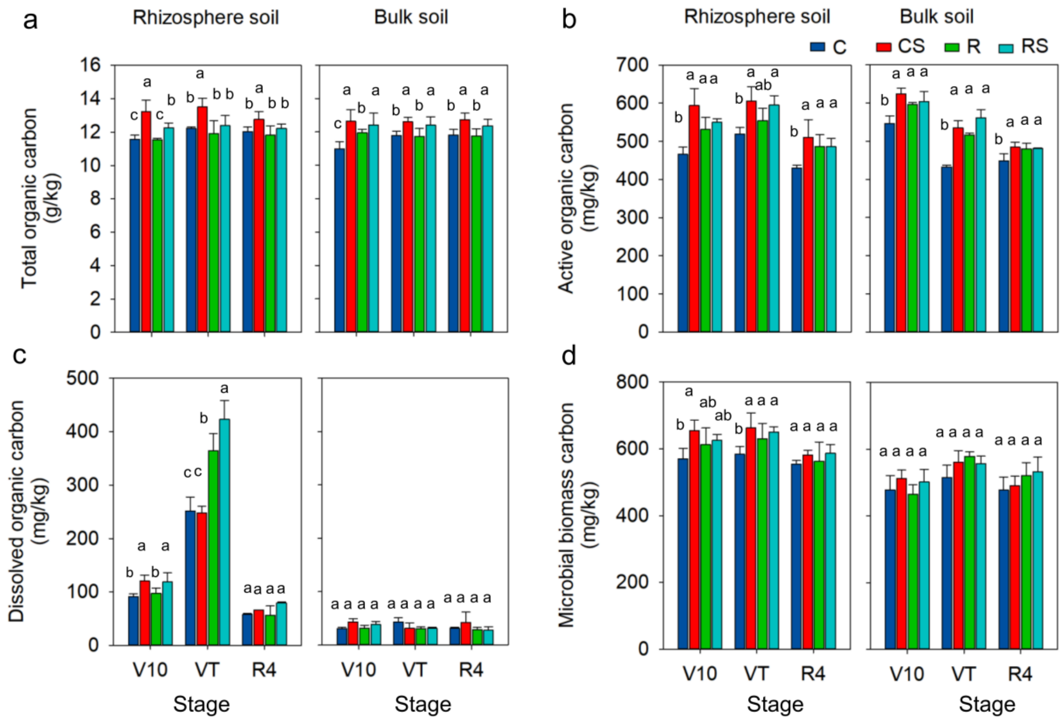

Fig.S1 Total organic carbon (a), active organic carbon (b), dissolved organic carbon (c) and microbial biomass carbon (d) in the four agricultural practices at three growth stages. Error bars indicate the standard deviation of three replicates. Different letters indicate significant differences ($P < 0.05$) between treatments at each growth stage.

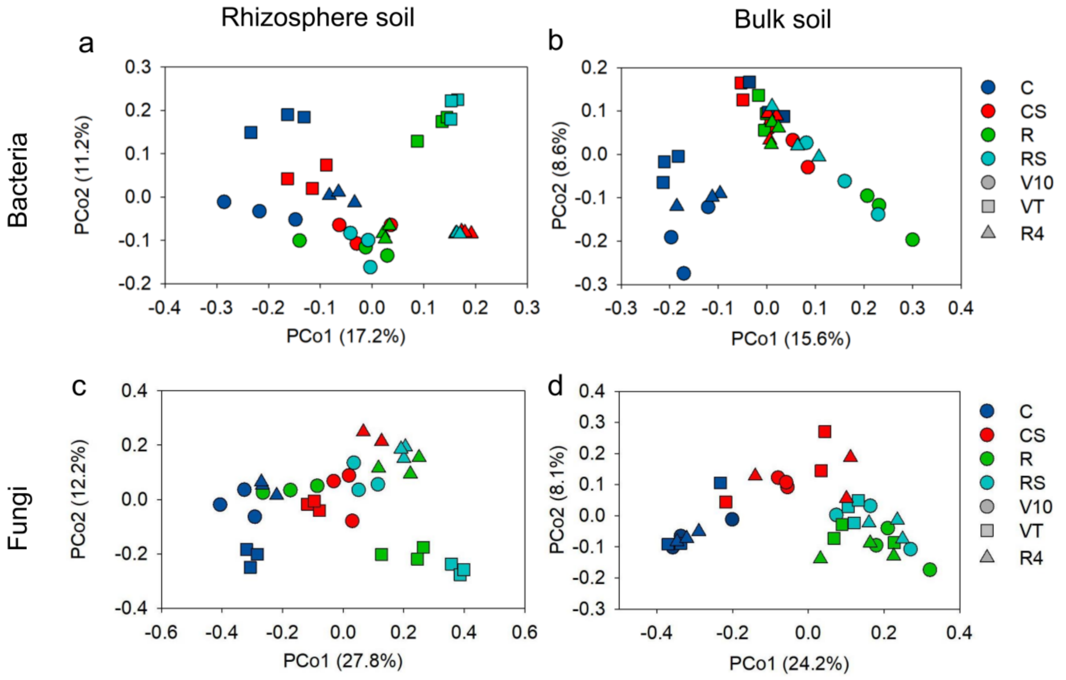

Fig.S2 PCoA of the bacteria community in rhizosphere soil (a) and bulk soil (b); PCoA of the fungi community in rhizosphere soil (c) and bulk soil (d). C: continuous maize cropping without straw retention; CS: continuous maize cropping with straw mulching; R: maize-peanut rotation without straw retention; RS: maize-peanut rotation with straw mulching; V10: the tenth leaf stage; VT: tassel stage; R4: dough stage.
